# Evaluation of the Effect of Momelotinib on Cardiac Repolarization: A Thorough QT Study

**DOI:** 10.1002/cpdd.1509

**Published:** 2025-01-23

**Authors:** Georgios Vlasakakis, Yolanda Alvarez, Timothy Hart, Yu Liu Ho, Catherine Ellis

**Affiliations:** ^1^ GSK Collegeville PA USA; ^2^ GSK London UK

**Keywords:** cardiac repolarization, momelotinib, myelofibrosis, pharmacokinetics, QT interval

## Abstract

A randomized, partially blinded, placebo‐controlled, crossover study in 48 healthy adults assessed the effect of momelotinib on the heart rate‐corrected QT interval (QTc) using the Fridericia formula (QTcF). QTc was evaluated for momelotinib 200 mg (therapeutic dose), momelotinib 800 mg (supratherapeutic dose), moxifloxacin 400 mg (positive control), and placebo. Pharmacokinetic profiles of momelotinib and its active metabolite M21 were evaluated. Momelotinib did not prolong QTcF, as the upper bounds of the 2‐sided 90% confidence intervals (CIs) for the mean difference between doses of momelotinib and placebo were <10 milliseconds at all time points. The lower limit of the 2‐sided 98% CI for the mean difference in QTcF between moxifloxacin versus placebo was >5 milliseconds at 2, 3, and 4 hours after dosing, demonstrating assay sensitivity. There was no positive relationship between momelotinib plasma concentrations and QTcF. Adverse events (AEs) were more frequent with the supratherapeutic dose of momelotinib, but none were considered severe. There were no deaths, serious AEs, or AEs leading to study discontinuation. Neither therapeutic nor supratherapeutic doses of momelotinib led to clinically significant effects on the QTc interval, supporting a negative finding for QTc prolongation from this thorough QT study.

Momelotinib is a potent oral inhibitor of Janus kinase 1 (JAK1)/JAK2/activin A receptor type 1 (ACVR1) that was first approved in the United States in September 2023, with subsequent approvals in Europe, Japan, and other regions for the treatment of intermediate‐ or high‐risk myelofibrosis (MF), including primary or secondary MF (post‐polycythemia vera and post‐essential thrombocythemia), in adults with anemia.^1^ Anemia and red blood cell transfusion dependence are important negative prognostic indicators in MF and are associated with diminished quality of life.^2^, ^3^, ^4^, ^5^, ^6^, ^7^ Momelotinib's inhibition of JAK‐signal transducer and activator of transcription signaling in MF blocks inflammatory cytokine production and clonal proliferation, leading to improvements in symptoms and splenomegaly, and its inhibition of ACVR1 and the subsequent decrease in hepcidin expression increases iron availability, thereby addressing iron‐restricted anemia associated with MF.^8^, ^9^, ^10^

Preclinical and clinical translational studies established this mechanistic explanation for the anemia benefits of momelotinib. Momelotinib's inhibitory potency against ACVR1 was determined in an enzymatic assay, with observed half‐maximal inhibitory concentration (IC_50_) values ranging from 6.8 to 8.4 nM.^8^ The active metabolite of momelotinib, M21,^11^ also inhibited JAK1/JAK2/ACVR1 in vitro, with a pharmacological activity index of approximately 40%.^10^ Both momelotinib and M21 potently bound ACVR1 in an adenosine triphosphate–independent competitive binding assay and reduced RNA levels of hepcidin in HepG2 hematoma cells.^8^, ^10^ Furthermore, in a rat model of anemia of chronic disease, momelotinib treatment normalized hemoglobin levels and red blood cell counts by direct inhibition of ACVR1, leading to a downstream decrease in suppressor of mothers against decapentaplegic (SMAD) phosphorylation and subsequent decrease in hepcidin expression.^8^ Consistent with these preclinical data, clinical translational results from a phase 2 study showed that momelotinib treatment reduced or reversed transfusion dependence in patients with MF in conjunction with decreased blood hepcidin levels and subsequent increased serum iron, hemoglobin, and erythropoiesis.^9^ Clinical results from phase 3 studies also demonstrated decreased transfusion requirement with momelotinib compared with trial comparators.^12^

The recommended momelotinib dose of 200 mg orally once daily is the approved optimal therapeutic dose in MF based on safety, efficacy, pharmacokinetic (PK), and pharmacodynamic results.^10^ Momelotinib undergoes rapid oral absorption with a time to achieve maximum plasma concentration (T_max_) of approximately 2 hours postdose, a steady‐state mean maximum plasma concentration (C_max_) of 479 ng/mL, a mean area under the curve from time 0 to the end of the dosing interval (AUC_tau_) of 3288 ng•h/mL, and a half‐life (t_½_) of approximately 4‐8 hours with minimal accumulation in plasma exposure following a 200‐mg clinical dose.^10^ Momelotinib exposures increase dose proportionally up to 300 mg but less than dose proportionally at higher doses.^10^

Prolongation of the QT interval, the electrocardiographic representation of the delay in cardiac repolarization, may result in the development of cardiac arrhythmias such as torsade de pointes and other ventricular arrhythmias.^13^ Because of this potential, evaluation of changes in QT interval has become a focus in drug development.^14^ Before clinical investigations, the potential for momelotinib to affect QTc interval was evaluated in vitro in a human Ether‐a‐go‐go Related Gene (hERG) assay and in vivo in conscious telemetered beagle dogs. These preclinical studies were negative for QTc prolongation, and a clinical thorough QT (TQT) study was conducted to further confirm the preclinical findings.

Here, we present a randomized, partially blinded, placebo‐ and positive‐controlled TQT study of 2 doses of momelotinib in healthy volunteers to evaluate the effects of momelotinib on QT/QTc interval prolongation.

## Methods

### Ethics

The protocol (GS‐US‐352‐1150) used in this analysis was approved by an institutional review board (Schulman Associates IRB; Sunrise, FL, USA) before the study started and was conducted in accordance with recognized international scientific and ethical standards, including, but not limited to, the ICH E6 guideline for Good Clinical Practice and the original principles embodied in the Declaration of Helsinki. All participants provided written informed consent for study participation before any procedures were performed.

### Study Design

This phase 1, single‐center (Seaview Research, Miami, FL, USA), partially blinded, randomized, placebo‐ and positive‐controlled, 4‐period single‐dose crossover study evaluated the effect of momelotinib on time‐matched change from baseline QTcF (corrected QT interval using the Fridericia formula) and explored the effect of momelotinib on other electrocardiogram (ECG) parameters. The objective of this TQT study was to evaluate the potential of momelotinib to prolong ventricular repolarization at therapeutic and supratherapeutic doses on time‐matched, baseline‐adjusted, placebo‐corrected QTcF using a study design administered at the clinic.

The therapeutic dose used for this study was the approved dose of momelotinib of 200 mg orally once daily. Momelotinib 800 mg once daily was considered appropriate to assess supratherapeutic exposure because exposure at this dose exceeded exposures observed in drug‐drug interactions studies and studies in organ‐impaired patients.^15^, ^16^

The study also explored the effect of momelotinib on individual‐specific QT interval correction (QTcI), which corrected QT calculation using individual correction, and other ECG parameters (eg, heart rate [HR], PR, QRS), determined the PK of momelotinib and its active metabolite M21, investigated the relationship between time‐matched, baseline‐adjusted, placebo‐corrected QTc and momelotinib plasma concentrations, and evaluated the safety and tolerability of momelotinib in healthy volunteers at the 2 administered doses.

Eligible participants were healthy male and nonpregnant, nonlactating female volunteers aged 18‐45 years with a body mass index from 19 to 30 kg/m^2^ at screening, normal kidney function (creatinine clearance ≥90 mL/min), and an ECG with normal PR and QT intervals or 1 with abnormalities that were considered clinically insignificant by the investigator in consultation with the sponsor. Participants were excluded if they had taken any prescription medication or over‐the‐counter medication, including herbal products, within 28 days of starting the study drug dosing except for vitamins, acetaminophen, ibuprofen, and/or hormonal contraceptive medication.

**Figure 1:**
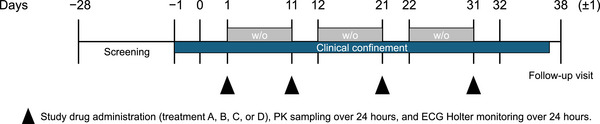
Study schema. Prospective participants were screened within 28 days before study drug dosing. Participants were admitted to the study clinic on day −1 and remained at the clinic until the morning of day 32. Participants completed 4 periods. Each period comprised 1 day of dosing with 1 of 4 study drug treatments. Periods 1 through 3 were followed by a washout period to avoid carryover effects. Following period 4 dosing, participants returned to the clinic 7 (±1) days after the last dose for an in‐clinic follow‐up visit. ECG, electrocardiogram; PK, pharmacokinetic; w/o, washout.

Participants received treatment on 4 days (1 day each for treatments A, B, C, and D) over the course of 31 days (Figure 1). All study treatments were administered orally at the study center in the morning at approximately the same time on each dosing day (days 1, 11, 21, and 31) with 240 mL of water following an overnight fast (no food or drink, except water, for ≥8 hours) and within 5 minutes of completing a standard moderate fat breakfast. Treatment A (therapeutic exposure) was a single oral dose of 200 mg momelotinib (1 × 200‐mg momelotinib tablet) plus 3 × placebo‐to‐match (PTM) momelotinib tablets, under fed conditions. Treatment B (supratherapeutic exposure) was a single oral dose of 800 mg momelotinib (4 × 200‐mg momelotinib tablets), under fed conditions. Treatment C (placebo control) was a single oral dose of 4 × PTM momelotinib tablets, under fed conditions. Treatment D (positive control) was a single oral dose of 400 mg moxifloxacin (1 × 400‐mg moxifloxacin tablet), under fed conditions. The sequence of treatments for each participant was randomized. Periods 1 through 3 were followed by a washout period to avoid carryover effects. Momelotinib in treatments A, B, and C and matching placebos in A and C were given in a blinded fashion, and the positive control, moxifloxacin in treatment D, was not blinded. Moxifloxacin 400 mg has been shown to reliably produce a 5‐ to 10‐milliseconds change in QTc duration after a single dose and was used for study validation and to assess assay sensitivity.^17^, ^18^

On days 1, 11, 21, and 31, time‐matched ECGs were collected in triplicate over a 24‐hour period using a digital 12‐lead Holter monitor (Mortara Instrument, Milwaukee, WI, USA) at the following time points: predose (baseline) at 1.5, 1, and 0.5 hours before the morning meal and postdose at 0.5, 1, 1.5, 2, 3, 4, 6, 8, 12, and 24 hours following administration of study drugs. A single ECG was collected at approximately 2 hours postdose for safety monitoring. Intensive sampling for PK assessments was also performed on days 1, 11, 21, and 31. All ECG acquisitions were completed before blood sample collections. Following period 4 dosing, participants returned to the clinic 7 (±1) days after the last dose for an in‐clinic follow‐up visit.

### Electrocardiography

ECG readings from lead II were used in all analyses. The same lead must have been utilized for analysis of the same participant across all treatment groups and time points. All reported QTc values were calculated as the mean of 3 replicate QTc measurements within 5 minutes leading up to each prespecified ECG collection time point. ECG‐derived QT intervals were corrected based on HR with Fridericia correction, QTcF = QT/RR^0.333^, as the primary method. QTcF is currently the standard adopted by the US Food and Drug Administration when submitting QT data for review. An individual correction (QTcI) was used as a supportive method. For the QTcI, all pairs of QT and RR intervals collected at predose baseline (from all treatments) and placebo treatment from each participant were used to determine an individual's correction coefficient following a linear regression model: log (QT_i_) = log (a_i_) + b_i_ log (RR_i_). The resulting slope (b_i_) for each participant was then used to calculate the correction for the QT intervals at each time point for that participant: QTcI_i_ = QT_i_/RR^bi^.

### Bioanalytic Procedures and Pharmacokinetic Analysis

Plasma concentrations of momelotinib, M21, and moxifloxacin were determined using a validated high‐performance liquid chromatography‐tandem mass spectrometry (HPLC‐MS/MS) bioanalytical method, with octadeuterated momelotinib, hexadeuterated M21, and tetradeuterated moxifloxacin as respective internal standards (Frontage Laboratories, Inc., Exton, PA, USA). Momelotinib and M21 were isolated from human plasma by solid‐phase extraction, with analyte separation achieved by reversed‐phase HPLC (Shimadzu HPLC pump and autosampler; Valco switch valve) with a 1 mL/min step gradient elution (mobile phase A: 5 mM aqueous ammonium acetate; mobile phase B: 5 mM ammonium acetate in methanol; vol:vol ratios and times were A:B 50:50 to 1.80 minutes, 10:90 to 2.00 minutes, 0:100 to 3.9 minutes, 0:100 at 2 mL/min to 4.00 minutes, and 50:50 at 1 mL/min to 5.40 minutes) on a YMC Basic column (50 × 4.6 mm, 3 µm). MS/MS detection was set at mass transitions of m/z 415.2→286.2 for momelotinib and 423.2→289.2 for its internal standard, and 429.2→345.2 for M21 and 435.2→351.2 for its internal standard, in positive mode. The calibrated range was 0.5‐1000 ng/mL. Analysis of spiked control samples demonstrated intra‐assay precision (percentage coefficient of variation) of 5.2% for momelotinib and 9.9% for M21; intra‐assay accuracy (percentage relative error) ranged from −9.2% to −6.2% and −8.7% to −6.2% for momelotinib and M21, respectively.^19^

PK parameters, including C_max_, the area under the curve from 0 to the last measurable concentration (AUC_last_), the area under the curve from 0 to infinity (AUC_inf_), T_max_, and t_½,_ were derived for momelotinib, M21, and moxifloxacin using WinNonlin (Phoenix WinNonlin Professional, version 6.3; Pharsight Corporation, Mountain View, CA, USA). Concentration values below the lower limit of quantitation (BLQ) were treated as 0.

### Safety Assessments

Safety was assessed through the reporting of adverse events (AEs; coded using Medical Dictionary for Regulatory Activities version 17), physical examinations and clinical laboratory test findings, 12‐lead safety ECGs, concomitant medications, vital signs determined at screening, periodic intervals during the inpatient period, and follow‐up.

### Statistical Analyses

A sample size of 40 evaluable participants provided 90% power to reject the null hypothesis that momelotinib prolonged the QTc interval by ≥10 milliseconds compared with placebo. This calculation was based on a 1‐sided testing procedure with a type I error rate of 5%, with assumptions that within‐participant standard deviation (SD) was 6.02 milliseconds (based on previous Gilead Sciences TQT studies), ECG measurements would be obtained at 10 postdose time points on each sampling day, and the true difference between study drug and placebo was 4.5 milliseconds. A 20% overage was built into the study sample size, thus requiring a total enrollment of 48 participants.

QTcF and the change from the predose baseline in QTcF were summarized using descriptive statistics by treatment group. A parametric (normal theory) analysis of covariance using a mixed‐effects model appropriate for a crossover design was fitted to the change from predose baseline in QTcF, including sequence, period, treatment, time point, treatment by time point interaction, and sex as fixed effects; participant within the sequence as a random effect; and the predose baseline QTcF as a continuous covariate. Momelotinib was concluded to have no QTcF prolongation effect if the upper bound of the 1‐sided 95% confidence interval (CI) for the mean difference between the test drug and placebo was <10 milliseconds for all postdose time points. A Bonferroni correction was used in constructing the CIs. A similar analysis was conducted for change from the predose baseline in QTcI, HR, PR, and QRS. Assay sensitivity was confirmed if the lower limit of the 2‐sided 98% CI for the mean difference between the positive control (moxifloxacin) and placebo was >5 milliseconds for ≥1 of the postdose prespecified time points. Categorical analyses of QTc evaluated the QTc interval with respect to the number and percentage of participants having a QTc interval increase from the predose baseline of >30 or >60 milliseconds by treatment group and the number and percentage of participants having absolute QTc interval prolongation of >450, >480, or >500 milliseconds at any postdose assessments but not at predose assessments by treatment group. Descriptive statistics and categorical analysis were conducted for other ECG parameters, including HR, PR, and QRS. Treatment‐emergent morphological ECG abnormalities were summarized (number and percentage) by treatment group.

The plasma concentrations of momelotinib, metabolite M21, and moxifloxacin were listed by the participant and summarized using descriptive statistics by treatment group at each protocol‐specific time point. Plasma concentrations of momelotinib, M21, and moxifloxacin were plotted over time in semilogarithmic and linear formats as mean ± SD and as median (Q1, Q3) for each treatment group.

A linear mixed‐effect model was used to quantify the relationship between plasma concentrations of momelotinib and M21 and ΔΔQTcF as well as ΔΔQTcI with sex as a fixed effect and participant as a random effect.

Safety data were listed by participant and summarized by treatment group using the frequency of event/abnormality or descriptive statistical summaries, as appropriate.

## Results

### Study Population

The study enrolled 48 healthy volunteers, all of whom were randomized, with 6 participants randomized to each treatment sequence. All participants received each study treatment (treatments A, B, C, and D), and no participants withdrew from the study.

There was a slightly higher percentage of men (58.3%) than women (41.7%). The median age was 36 years (range, 18‐45 years), and the majority of participants were White (87.5%) (Table S1).

### Evaluation of Assay Sensitivity

**Figure 2:**
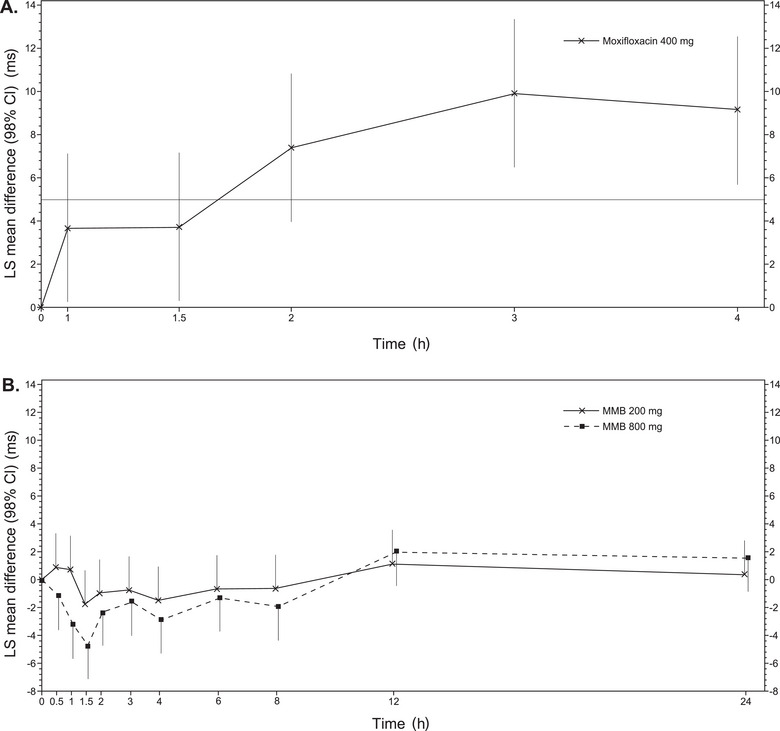
Evaluation of assay sensitivity of QTcF. (A) Change in QTcF after treatment with moxifloxacin 400 mg (positive control) was used to evaluate assay sensitivity. (B) Change in QTcF after treatment with 200 and 800 mg of momelotinib. LS means and CIs were based on the mixed‐effect model, including sequence, period, treatment, time point, treatment by time point interaction, and sex as fixed effects; participants with the sequence as a random effect; and the predose baseline QTcF as a continuous covariate. CI, confidence interval; LS, least squares; MMB, momelotinib; QTcF, corrected QT interval using the Fridericia formula.

Moxifloxacin 400 mg was used as a positive control to evaluate the assay sensitivity for time‐matched, baseline‐adjusted, placebo‐corrected QTcF and QTcI. The lower bound of the 2‐sided 98% CI for the mean difference between the positive control and placebo was >5 milliseconds at 2 time points (3 and 4 hours) after dosing, thereby establishing assay sensitivity (Figure 2A and Table S2).

### Electrocardiography Analysis After Momelotinib Treatment

Momelotinib was concluded to have no QTcF prolongation effect, as the upper bound of the 2‐sided 90% CIs for the mean difference between therapeutic or supratherapeutic doses of momelotinib and placebo was <10 milliseconds at all time points after dosing (Figure 2B and Table S3). Small negative changes in QTcF were observed at both doses. Results from the analysis of QTcI were consistent with those from the analysis of QTcF (Figure S1). No participants had a QTcF interval change from predose baseline of >30 or >60 milliseconds at any time point during any treatment (Table S4). Treatment‐emergent absolute QTcF intervals of >480 or >500 milliseconds were not observed for any participants following any treatment. Three participants had treatment‐emergent absolute QTcF intervals of >450 milliseconds following moxifloxacin treatment. One participant had an increase in QTcI of >30 milliseconds from predose baseline following momelotinib 800 mg. Treatment‐emergent absolute QTcI intervals of >480 or >500 milliseconds were not observed in any participants following any treatment. Maximal mean changes from baseline in PR interval, QRS deflection, RR interval, and HR were comparable among all treatments (including momelotinib 200 and 800 mg, placebo, and moxifloxacin) (Table S5). No treatment‐emergent U‐wave abnormalities were observed, and treatment‐emergent T‐wave abnormalities were considered not clinically significant (Table S4).

### Pharmacokinetics of Momelotinib and M21

**Figure 3:**
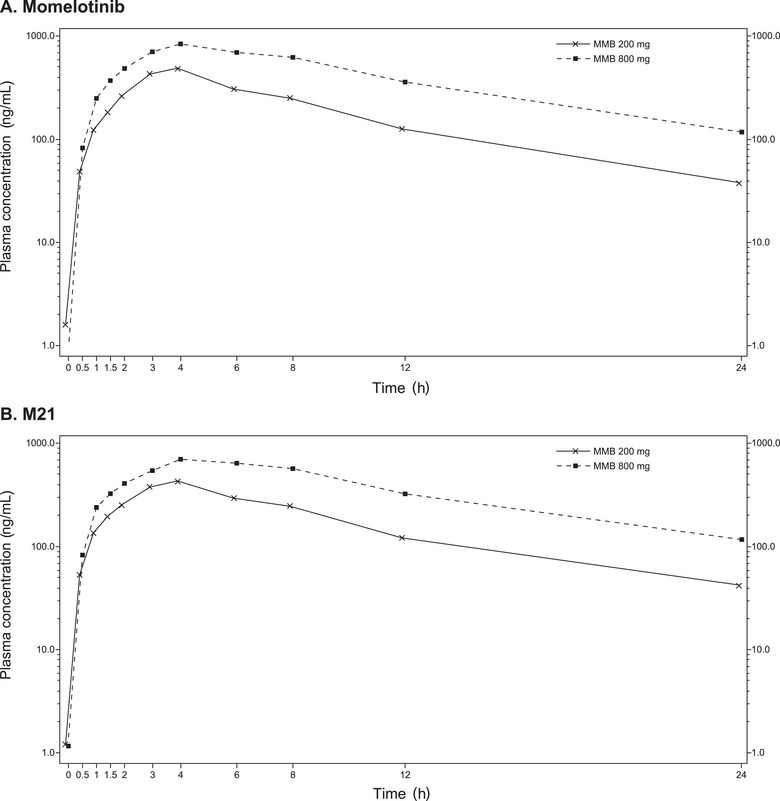
Pharmacokinetics of momelotinib and M21. Mean (SD) plasma concentration‐time profile for momelotinib (A) and M21 (B) after a single oral dose of 200 or 800 mg of momelotinib. MMB, momelotinib; SD, standard deviation.

Momelotinib and M21 exposures were expectedly higher at 800 versus 200 mg and less than proportional. Momelotinib at 200‐ and 800‐mg doses had a median T_max_ of 3.5 and 4 hours under fed conditions and a median terminal t_½_ of 5 and 6 hours, respectively (Table 1). Momelotinib AUC_inf_ and AUC_last_ were approximately 2.4‐fold higher and C_max_ was approximately 1.8‐fold higher for the 800‐mg than the 200‐mg momelotinib dose (4‐fold dose range) (Figure 3A and Table S6). M21 AUC_inf_ and AUC_last_ were approximately 2.2‐fold higher and C_max_ was approximately 1.8‐fold higher for the 800‐mg than the 200‐mg momelotinib dose (Figure 3B and Table S6). PK parameters across the study doses and dose proportionality results were consistent with historical data.^19^ PK results of moxifloxacin in this analysis were consistent with previously published data (Table S7).^20^

**Table 1:** Pharmacokinetics of Momelotinib and M21

PK parameter^a^	Momelotinib 200 mg (N = 48)	Momelotinib 800 mg (N = 48)	GLSM ratio	90% CI
**Momelotinib**				
AUC_inf_ (h•ng/mL)	4368 (61.7)	10,545 (63.7)	239	221‐258
AUC_last_ (h•ng/mL)	3960 (54.4)	9146 (54.9)	231	214‐249
C_max_ (ng/mL)	515 (43.9)	906 (38.3)	180	167‐194
t_1/2_ (hour)	5.7 (2.0)	6.5 (2.2)	‐	‐
**M21**				
AUC_inf_ (h•ng/mL)	4236 (34.2)	9615 (37.5)	225	210‐241
AUC_last_ (h•ng/mL)	3711 (37.8)	8011 (39.3)	217	203‐232
C_max_ (ng/mL)	430 (42.4)	739 (39.2)	176	163‐190
t_1/2_ (hour)	7.0 (2.8)	7.3 (2.5)	‐	‐

Statistical analysis for dose proportionality was based on the mixed‐effect model, including sequence, period, and treatment as fixed effects and participants within the sequence as a random effect.

AUC_inf_, the area under the curve from 0 to infinity; AUC_last_, the area under the curve from 0 to the last measurable concentration; CI, confidence interval; C_max_, maximum plasma concentration; CV, coefficient of variation; GLSM, geometric least‐squares mean; PK, pharmacokinetic; t_1/2_, terminal elimination half‐life.

^a^t_1/2_ (hour) is presented as mean (SD); all other parameters are mean (% CV).

### Pharmacokinetic and Pharmacodynamic Relationship

**Figure 4:**
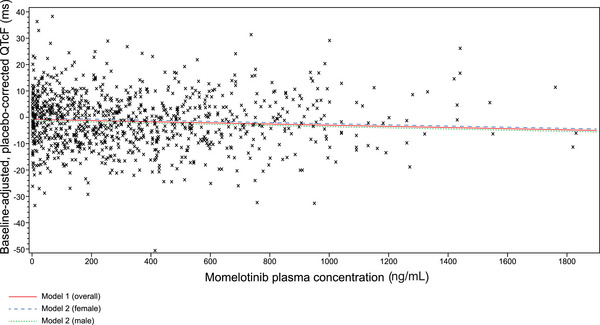
Time‐matched, baseline‐adjusted, and placebo‐corrected QTcF versus momelotinib plasma concentrations. A scatter plot depicting the relationship between ΔΔQTcF and momelotinib plasma concentrations using a linear mixed‐effect model with sex as a fixed effect and participant as a random effect. For momelotinib 200 mg, mean maximum concentration was 515, predicted CCHG_QTcF was −1.27, and the upper 95% 1‐sided CI was 0.51. For momelotinib 800 mg, mean maximum concentration was 902, predicted CCHG_QTcF was −2.31, and the upper 95% 1‐sided CI was −0.56. For both Models 1 (overall; ΔΔQTcF = −0.54 + −0.002*[momelotinib concentration in ng/mL]) and 2 (by sex; ΔΔQTcF = −1.09 + −0.002*[momelotinib concentration in ng/mL] + [0.94 if female]), conditional R^2^ was 0.26.^31^ CCHG_QTcF, time‐matched, baseline‐adjusted, and placebo‐corrected QTcF; CI, confidence interval; QTcF, corrected QT interval using the Fridericia formula.

There were no clinically relevant relationships between time‐matched, baseline‐adjusted, placebo‐corrected QTcF and plasma concentrations of momelotinib (Figure 4 and Table S8) or M21 (Figure S2). Although the effect of momelotinib concentration was statistically significant (overall regression, *P* = .38; regression with sex as a fixed effect, *P* = .037) the small, negative slope (−0.002) suggested that there were no clinically relevant relationships between momelotinib plasma concentration and ΔΔQTcF interval. There was no significant relationship between QTcF and M21 concentration (overall regression, *P* = .44; regression with sex as a fixed effect, *P* = .43).

### Safety Results

There were no deaths, serious AEs, or discontinuations due to AEs during the study. The most frequently reported treatment‐emergent AEs by treatment were as follows: momelotinib 200 mg, headache (10.4%, 5 participants), dizziness (4.2%, 2 participants), and metrorrhagia (4.2% overall, 10% among women, 2 participants), with all other events occurring in 1 participant each; momelotinib 800 mg, headache (20.8%, 10 participants), nausea (16.7%, 8 participants), and dizziness and flushing (12.5%, 6 participants each); placebo, all events occurring in 1 participant each; and moxifloxacin, metrorrhagia (4.2% overall, 10% among women, 2 participants), with all other events occurring in 1 participant each (Table S9).

All AEs in momelotinib‐treated participants were mild in severity except for a moderate acneiform dermatitis in 1 participant in the momelotinib 800‐mg group, and all treatment‐related AEs resolved. The only severe treatment‐emergent laboratory abnormality was a grade 3 increased triacylglycerol lipase in 1 placebo‐treated participant, the incidences of mild or moderate laboratory abnormalities were similar across treatments. No notable differences between treatments in the incidences of specific laboratory abnormalities were observed. Mean changes from predose vital signs (temperature, pulse, systolic blood pressure, diastolic blood pressure, and respiration rate) were small in magnitude and appeared to be similar between groups.

## Discussion

This phase 1 clinical study found that therapeutic and supratherapeutic doses of momelotinib did not change the QTc interval in healthy participants. Based on the results of this study, momelotinib regulatory labeling notes that there is no clinically relevant effect on QTc.

Exposures of momelotinib and its active metabolite M21 at the 2 doses studied were slightly less than proportional to the dose and consistent with data from a relative bioavailability study.^19^ Exposures of momelotinib and M21 achieved in this study are deemed to adequately represent therapeutic and supratherapeutic exposures and are consistent with previously reported PK results of the therapeutic 200‐mg momelotinib dose in healthy participants.^15^, ^19^ There were no clinically relevant relationships between time‐matched, baseline‐adjusted, placebo‐corrected QTcF or QTcI and plasma concentrations of momelotinib or M21. No severe or serious AEs or discontinuations due to AEs were reported in healthy participants treated with momelotinib at either dose.

The data are consistent with previous preclinical and clinical evaluations of momelotinib effects on ECG parameters, which showed no clinically meaningful QT interval prolongation. Momelotinib at concentrations up to 10 µM and a positive control article (cisapride) were applied for up to 8 minutes to HEK293 cells expressing human recombinant hERG channels. Momelotinib was a weak inhibitor of the hERG channel K+ current with an IC_50_ of >10 µM, which is 45‐fold higher than the estimated free drug C_max_ (0.22 µM) at the mean momelotinib C_max_ of 479 ng/mL for a therapeutic 200‐mg dose. The QTc interval was evaluated in conscious, instrumented, telemetered male beagle dogs (n = 4) following single oral doses of vehicle (acidified water) or 5, 30, or 100 mg/kg of momelotinib using a Latin square design. Momelotinib at 100 mg/kg produced a marked decrease in arterial blood pressure with a concurrent increase in HR, along with the subsequent related changes to the lead II ECG parameters. The QTc intervals were not notably altered by momelotinib. Momelotinib C_max_ (1913 ng/mL total; 367 ng/mL free) in male dogs administered 100 mg/kg was approximately 4‐fold higher than the estimated free drug C_max_ of 92 ng/mL in patients with MF who received the recommended dose of 200 mg daily and 2‐fold higher than those who received the 800‐mg supratherapeutic dose. These nonclinical results suggested that momelotinib was unlikely to prolong the QT interval in patients, as confirmed in the present study.

In pivotal phase 3 studies, patients with QTc intervals of >450 millisecods (in SIMPLIFY‐1 and SIMPLIFY‐2) and >500 milliseconds (in MOMENTUM), unless attributed to bundle branch block, were excluded. Based on the results from this QTc study and review of cardiovascular‐related events, investigators decided that there were no safety concerns with stopping the active monitoring of ECG, and ECG monitoring was discontinued in SIMPLIFY‐1 and SIMPLIFY‐2. Among 725 patients in a long‐term pooled safety analysis of SIMPLIFY‐1, SIMPLIFY‐2 (before discontinuation of monitoring), and MOMENTUM, 4 had QT prolongation, but only 1 patient in SIMPLIFY‐1 had a recorded grade 3 (≥500 milliseconds) QTc prolongation that led to discontinuation of momelotinib.^21^, ^22^, ^23^, ^24^

QTc prolongation is not a class effect of JAK inhibitors. TQT evaluations of ruxolitinib and fedratinib, other JAK inhibitors approved to treat MF, concluded that there was no significant QT prolongation effect, similar to momelotinib.^25^, ^26^ However, the JAK inhibitor pacritinib, approved to treat MF in patients with platelet counts of <50×10^9^/L, carries a warning to avoid in patients with a baseline QTc of >480 milliseconds and in those who would require concomitant use of another drug with significant potential for QTc prolongation.^27^ These warnings were based on the higher incidence of QTc prolongations of >500 milliseconds with pacritinib compared with control.^28^ The chemical structure of pacritinib may explain the difference in QTc response: pacritinib is a cyclic ether, and ethers appear more frequently in QT‐prolonging drugs compared with drugs with no QT concerns.^29^ Since patients with MF are often older and have comorbidities requiring concurrent treatment with medications that may prolong QT intervals,^30^ selecting treatment with no QT prolongation may be especially important in this patient population.

Although the study population was younger and healthier than those who would typically receive momelotinib for treatment of MF, the use of healthy participants reduces variability in QTc interval measurements, and in population PK analysis, age was not a significant covariate. Therefore, the clinical exposures reported here are representative of patients with MF.

## Conclusion

Momelotinib administered at 200‐mg (therapeutic) and 800‐mg (supratherapeutic) doses and its active metabolite M21 showed no QTc prolongation liability in healthy adults. There was no clinically relevant relationship between plasma concentrations of momelotinib (or M21) and QTcF or QTcI. Thus, this is a negative TQT study demonstrating that neither therapeutic nor supratherapeutic doses of momelotinib were associated with significant ECG or wave morphology changes.

## Conflicts of Interest

G.V., Y.A., T.H., and C.E. are employees of and stockholders in GSK. Y.L.H. is currently an employee of GSK and a former employee of Gilead Science and owns Gilead stock.

## Funding

The manuscript was funded by GSK.

## Supporting information



Supporting Information
