# Supplementary material for: Evaluation of the Effect of Momelotinib on Cardiac Repolarization: A Thorough QT Study
Source: Clin Pharmacol Drug Dev. 2025 Jan 23;14(4):333–42. doi: 10.1002/cpdd.1509 (PMC11975203; doi:10.1002/cpdd.1509)
Supplement: Supplementary file 1 — Supporting Information [file CPDD-14-333-s001.docx]

# Supplemental Material

## Figures

**Figure S1. Noninferiority evaluation for time-matched, baseline-adjusted, and placebo-controlled QTcI.** CI, confidence interval; LS, least squares; QTcI, individual-specific QT interval correction.


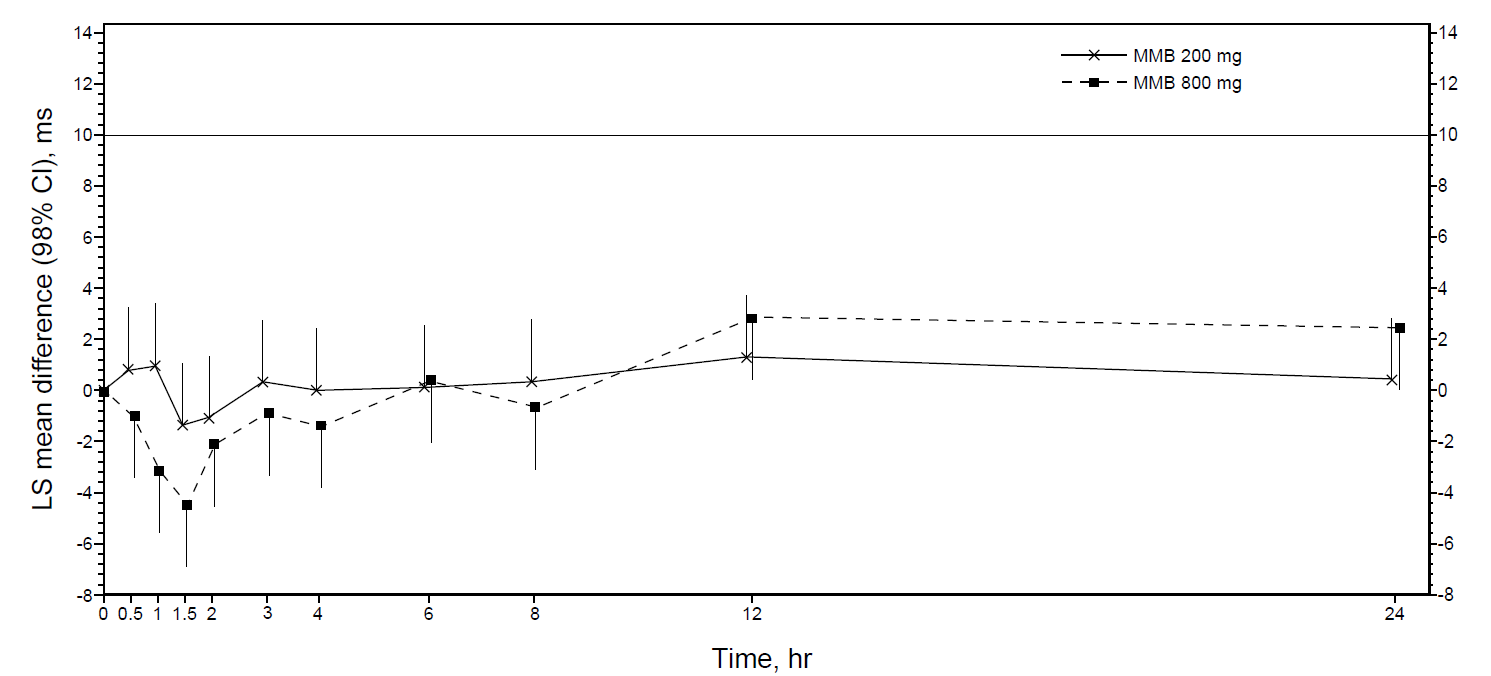


|  | LS means | | | Treatment difference | | 90% CI | |
| --- | --- | --- | --- | --- | --- | --- | --- |
| Scheduled time post dose, h | Momelotinib  200 mg  (N=48) | Momelotinib  800 mg  (N=48) | Placebo  (N=48) | Momelotinib  200 mg - Placebo | Momelotinib  800 mg - Placebo | Momelotinib  200 mg - Placebo | Momelotinib  800 mg - Placebo |
| 0.5 | −6.8 | −8.6 | −7.6 | 0.8 | −1.0 | −1.6 to 3.3 | −3.5 to 1.5 |
| 1 | −8.0 | −12.1 | −8.9 | 0.9 | −3.1 | −1.5 to 3.4 | −5.6 to −0.7 |
| 1.5 | −9.4 | −12.5 | −8.1 | −1.4 | −4.5 | −3.8 to 1.1 | −7.0 to −2.0 |
| 2 | −12.3 | −13.4 | −11.3 | −1.1 | −2.1 | −3.5 to 1.4 | −4.6 to 0.3 |
| 3 | −10.0 | −11.2 | −10.4 | 0.3 | −0.9 | −2.1 to 2.8 | −3.3 to 1.6 |
| 4 | −6.0 | −7.4 | −6.0 | 0.0 | −1.4 | −2.5 to 2.5 | −3.8 to 1.1 |
| 6 | −5.3 | −5.1 | −5.5 | 0.1 | 0.4 | −2.3 to 2.6 | −2.1 to 2.8 |
| 8 | −1.2 | −2.2 | −1.5 | 0.4 | −0.6 | −2.1 to 2.8 | −3.1 to 1.8 |
| 12 | −4.1 | −2.5 | −5.4 | 1.3 | 2.9 | −1.2 to 3.8 | 0.4 to 5.3 |
| 24 | −2.5 | −0.4 | −2.9 | 0.4 | 2.5 | −2.1 to 2.9 | 0.0 to 5.0 |

LS means and CI were based on the mixed-effect model, including sequence, period, treatment, time point, treatment by time point interaction, and sex as fixed effects; participants with sequence as a random effect; and the predose baseline QTcI as continuous covariate.

**Figure S2. Time-matched, baseline-adjusted, and placebo-corrected QTcF vs plasma concentrations of momelotinib metabolite (M21).** For momelotinib 200 mg, mean maximum concentration was 429, predicted CCHG_QTcf was −0.62, and the upper 95% 1-sided CI was 1.12. For momelotinib 800 mg, mean maximum concentration was 739, predicted CCHG_QTcf was −1.57, and the upper 95% 1-sided CI was 0.15. For Model 1 (overall; ΔΔQTcF = −0.96 + −0.00096*[momelotinib concentration in ng/mL]), conditional R^2^ was 0.25; for Model 2 (by sex; ΔΔQTcF = −1.51 + −0.00099*[momelotinib concentration in ng/mL] + [0.96 if female]), conditional R^2^ was 0.26.^1^ CCHG_QTcF, time-matched, baseline-adjusted, and placebo-corrected QTcF; CI, confidence interval; PK, pharmacokinetic; QTcF, corrected QT interval using the Fridericia formula.

**
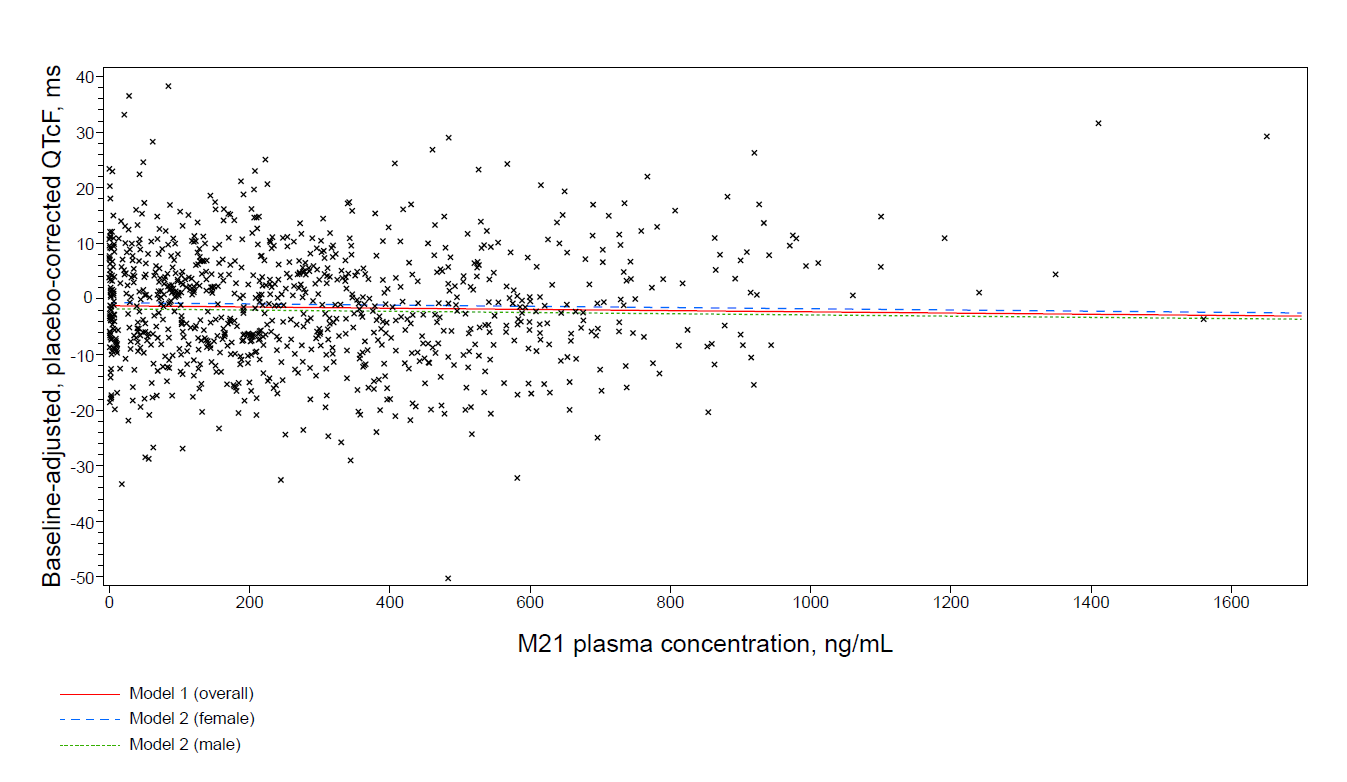
**

|  | | | 95% CI | |  |
| --- | --- | --- | --- | --- | --- |
|  | Estimate | Standard error | Lower | Upper | *P* value |
| Overall regression^a^ |  |  |  |  |  |
| Intercept (a) | −0.961 | 0.890 | −2.75 | 0.829 | .286 |
| Concentration (b) | −0.000960 | 0.00124 | −0.00340 | 0.00148 | .440 |
| Regression with sex as fixed effect^b^ |  |  |  |  |  |
| Intercept (a) | −1.51 | 1.31 | −4.15 | 1.12 | .253 |
| Concentration (b) | −0.000992 | 0.00124 | −0.00343 | 0.00145 | .426 |
| Sex (c): 0 = male; 1 = female | 0.963 | 1.66 | −2.37 | 4.30 | .564 |

^a^ Overall regression equation was CCHG_QTcF = a + (b × concentration). Overall PK/pharmacodynamic regression included concentration as a continuous covariate and participant within sequence as a random effect. ^b^ Regression equation with sex as a fixed effect was CCHG_QTcF = a + (b × concentration) + (c × sex). PK/pharmacodynamic regression with sex as a fixed effect included sex as a fixed effect, concentration as a continuous covariate, and participant as a random effect.

## Tables

**Table S1. Demographic and baseline characteristics**

| Characteristics | Safety and pharmacodynamic analysis set  (N=48) |
| --- | --- |
| Sex, n (%) |  |
| Male | 28 (58.3) |
| Female | 20 (41.7) |
| Age, years |  |
| Mean (SD) | 35 (7.7) |
| Median (range) | 36 (18-45) |
| Race, n (%) |  |
| White | 42 (87.5) |
| Black or African American | 6 (12.5) |
| Other | 0 |
| BMI, kg/m^2^ |  |
| Mean (SD) | 26.5 (2.1) |
| Median (range) | 26.8 (20.9-30.4) |
| Estimated GFR, mL/min ^a^ |  |
| Mean (SD) | 132.8 (23.8) |
| Median (range) | 131 (89.8-210.5) |

BMI, body mass index; GFR, glomerular filtration rate; SD, standard deviation.

^a^ Estimated GFR was estimated using the Cockcroft-Gault equation: (140 – Age [years] at Collection) × Weight (kg) × (0.85 if female) / (Creatinine [mg/dL] × 72).

##

**Table S2. Mixed-model analysis of change from predose baseline in QTcF (ms) for assay sensitivity evaluation**

|  | LS means | | Treatment difference | 98% CI |
| --- | --- | --- | --- | --- |
| Scheduled time post dose, h | Moxifloxacin  (N=48) | Placebo  (N=48) | Moxifloxacin-placebo | Moxifloxacin-placebo |
| 0.5 | −8.4 | −10.0 | 1.5 |  |
| 1 | −7.9 | −11.6 | 3.7 | 0.2-7.1 |
| 1.5 | −6.5 | −10.3 | 3.7 | 0.3-7.2 |
| 2 | −6.1 | −13.5 | 7.4 | 3.9-10.9 |
| 3 | −1.9 | −11.8 | 9.9 | 6.4-13.4 |
| 4 | 2.1 | −7.1 | 9.2 | 5.7-12.7 |
| 6 | −0.2 | −7.3 | 7.1 |  |
| 8 | 1.3 | −2.8 | 4.1 |  |
| 12 | −2.5 | −7.4 | 4.9 |  |
| 24 | 0.7 | −4.3 | 4.9 |  |

CI, confidence interval; LS, least squares; QTcF, corrected QT interval using the Fridericia formula.

CI and LS means were based on the mixed-effect model, including sequence, period, treatment, time point, treatment by time point interaction, and sex as fixed effects; participants with sequence as a random effect; and the predose baseline QTcF as a continuous covariate. Assay sensitivity analysis was performed only at postdose time points: 1, 1.5, 2, 3, and 4 h.

**Table S3. Mixed-model analysis of change from predose baseline in QTcF for noninferiority evaluation**

|  | LS means | | | Treatment difference | | 90% CI | |
| --- | --- | --- | --- | --- | --- | --- | --- |
| Scheduled time post dose, h | Momelotinib  200 mg  (N=48) | Momelotinib  800 mg  (N=48) | Placebo  (N=48) | Momelotinib  200 mg - placebo | Momelotinib  800 mg - placebo | Momelotinib  200 mg - placebo | Momelotinib  800 mg - placebo |
| 0.5 | −9.1 | −11.2 | −10.0 | 0.9 | −1.2 | (−1.6 to 3.3) | (−3.7 to 1.2) |
| 1 | −10.9 | −14.9 | −11.6 | 0.7 | −3.3 | (−1.8 to 3.2) | (−5.7 to−0.8) |
| 1.5 | −12.1 | −15.0 | −10.3 | −1.8 | −4.8 | (−4.2 to 0.7) | (−7.2 to 2.3) |
| 2 | −14.5 | −15.9 | −13.5 | −1.0 | −2.4 | (−3.4 to 1.5) | (−4.8 to 0.1) |
| 3 | −12.5 | −13.4 | −11.8 | −0.8 | −1.6 | (−3.2 to 1.7) | (−4.1 to 0.8) |
| 4 | −8.6 | −10.0 | −7.1 | −1.5 | −2.9 | (−4.0 to 1.0) | (−5.4 to −0.4) |
| 6 | −8.0 | −8.6 | −7.3 | −0.7 | −1.3 | (−3.1 to 1.8) | (−3.8 to 1.1) |
| 8 | −3.4 | −4.7 | −2.8 | −0.7 | −1.9 | (−3.1 to 1.8) | (−4.4 to 0.5) |
| 12 | −6.3 | −5.4 | −7.4 | 1.1 | −2.0 | (−1.3 to 3.6) | (−0.5 to 4.4) |
| 24 | −3.9 | −2.7 | −4.3 | 0.4 | −1.5 | (−2.1 to 2.8) | (−0.9 to 4.0) |

CI, confidence interval; LS, least squares; QTcF, corrected QT interval using the Fridericia formula.

CI and LS means were based on the mixed-effect model, including sequence, period, treatment, time point, treatment by time point interaction, and sex as fixed effects; participants with sequence as a random effect; and the predose baseline QTcF as a continuous covariate.

**Table S4. Categorical analysis for QTcF and ECG findings by treatment**

| Categorical analysis for QTcF by treatment^a^ | | | | |
| --- | --- | --- | --- | --- |
| n (%) | Momelotinib 200 mg (N=48) | Momelotinib 800 mg (N=48) | Placebo  (N=48) | Moxifloxacin 400 mg (N=48) |
| Observed value | | | | |
| >500 ms | 0 | 0 | 0 | 0 |
| >480 ms | 0 | 0 | 0 | 0 |
| >450 ms | 0 | 0 | 0 | 3 (6.3) |
| Change from predose/baseline | | | | |
| >60 ms | 0 | 0 | 0 | 0 |
| >30 ms | 0 | 0 | 0 | 0 |
| ECG findings^b^ |  |  |  |  |
| First degree AV block | |  |  |  |
| Predose/baseline | 0 | 1 (2.1) | 0 | 0 |
| Post dose | 0 | 1 (2.1) | 0 | 0 |
| Voltage criteria for LVH |  |  |  |  |
| Predose/baseline | 0 | 1 (2.1) | 1 (2.1) | 1 (2.1) |
| Post dose | 1 (2.1) | 1 (2.1) | 1 (2.1) | 1 (2.1) |
| Nonspecific ST-segment depression |  |  |  |  |
| Predose/baseline | 0 | 0 | 0 | 0 |
| Post dose | 0 | 0 | 1 (2.1) | 0 |
| Nonspecific ST-T segment abnormality |  |  |  |  |
| Predose/baseline | 0 | 0 | 0 | 0 |
| Post dose | 0 | 2 (2.4) | 0 | 0 |
| Nonspecific T-wave abnormality |  |  |  |  |
| Predose/baseline | 4 (8.3) | 0 | 5 (10.4) | 2 (4.2) |
| Post dose | 10 (20.8) | 15 (31.3) | 9 (18.8) | 9 (18.8) |
| Nonspecific T-wave abnormality with U wave |  |  |  |  |
| Predose/baseline | 0 | 0 | 0 | 0 |
| Post dose | 0 | 0 | 1 (2.1) | 0 |
| Nonspecific T-wave abnormality with U waves |  |  |  |  |
| Predose/baseline | 0 | 0 | 0 | 0 |
| Post dose | 0 | 0 | 1 (2.1) | 0 |
| Sinus arrhythmia |  |  |  |  |
| Predose/baseline | 3 (6.3) | 1 (2.1) | 1 (2.1) | 3 (6.3) |
| Post dose | 4 (8.3) | 1 (2.1) | 3 (6.3) | 2 (4.2) |
| Atrial premature complex(es) |  |  |  |  |
| Predose/baseline | 1 (2.1) | 0 | 0 | 1 (2.1) |
| Post dose | 2 (4.2) | 0 | 0 | 0 |
| Significant artifact |  |  |  |  |
| Predose/baseline | 0 | 0 | 0 | 0 |
| Post dose | 1 (2.1) | 2 (4.2) | 1 (2.1) | 1 (2.1) |
| Ventricular premature complex(es) |  |  |  |  |
| Predose/baseline | 0 | 0 | 1 (2.1) | 1 (2.1) |
| Post dose | 0 | 0 | 0 | 0 |

AV, atrioventricular; ECG, electrocardiogram; LVH, left ventricular hypertrophy; QTc, corrected QT interval; QTcF, corrected QT interval using the Fridericia formula.

^a^ Only participants with treatment-emergent QTc prolongation (>450-480, >480-500, and >500 ms) were counted as events for “Observed value” and included in the numerator. Treatment emergent means a participant had a QTc interval prolongation at any postdose assessment. ^b^ Only categories with morphological findings were included.

**Table S5. Descriptive summaries for ECG PR interval, QRS deflection, RR interval, and HR**

| **PR (msec)** | **Momelotinib 200 mg**  **(N=48)** | | **Momelotinib 800 mg**  **(N=48)** | |
| --- | --- | --- | --- | --- |
|  | **Mean (SD)** | **Median (range)** | **Mean (SD)** | **Median (range)** |
| **Predose** | 167 (18.7) | 164 (132.9-213.2) | 169 (18.6) | 166 (135.8-217.2) |
| **Post dose 0.5 h**  **Change from predose** | 164 (17.7) **^a^**  −2.0 | 163 (129.0-201.7) **^a^**  0.0 | 165 (17.9) **^a^**  −2.5 | 165 (136.0-205.3) **^a^**  −3.3 |
| **Post dose 1 h**  **Change from predose** | 162 (16.9) **^a^**  −3.7 | 160 (130.7-196.7) **^a^**  −2.2 | 164 (17.2)  −4.7 | 166 (134.0-208.0)  −3.2 |
| **Post dose 1.5 h**  **Change from predose** | 162 (18.3)  4.8 | 163 (128.0-197.3)  −5.4 | 162 (17.3)  −6.9 | 163 (128.0-202.0)  −6.5 |
| **Post dose 2 h**  **Change from predose** | 161 (17.4)  −5.7 | 162 (123.7-199.3)  −2.6 | 161 (17.2)  −7.3 | 162 (130.0-201.0)  −6.8 |
| **Post dose 3 h**  **Change from predose** | 162 (18.3) **^a^**  −5.5 | 161 (130.7-199.7) **^a^**  −4.7 | 160 (17.6)  −8.3 | 160 (130.0-193.0)  −7.4 |
| **Post dose 4 h**  **Change from predose** | 162 (18.3)  −5.4 | 161 (126.7-209.0)  −4.6 | 159 (17.4)  −9.1 | 159 (130.0-196.7)  −7.7 |
| **Post dose 6 h**  **Change from predose** | 159 (18.6)  −8.4 | 159 (128.7-200.7)  −6.8 | 159 (17.4)  −10.0 | 157 (128.0-197.3)  −9.0 |
| **Post dose 8 h**  **Change from predose** | 158 (19.4)  −8.6 | 158 (124.3-204.3)  −8.0 | 160 (17.0)  −8.7 | 158 (126.3-200.3)  −7.2 |
| **Post dose 12 h**  **Change from predose** | 161 (18.1)  −6.2 | 160 (130.3-203.0)  −6.3 | 163 (18.5) | 164 (135.0-209.0)  −4.7 |
| **Post dose 24 h**  **Change from predose** | 166 (17.6)  −1.3 | 165 (132.3-198.7)  −0.2 | 168 (19.8) | 166 (131.3-209.3)  −0.6 |

| **PR (msec)** | **Placebo**  **(N=48)** | | **Moxifloxacin 400 mg**  **(N=48)** | |
| --- | --- | --- | --- | --- |
|  | **Mean (SD)** | **Median (range)** | **Mean (SD)** | **Median (range)** |
| **Predose** | 167 (18.4) | 162 (130.1-203.4) | 166 (18.2) | 165 (132.4-200.0) |
| **Post dose 0.5 h**  **Change from predose** | 163 (18.3)  −4.0 | 159 (134.3-196.7)  −2.7 | 165 (18.5)  −0.7 | 165 (133.3-206.7)  −0.8 |
| **Post dose 1 h**  **Change from predose** | 162 (17.9) −5.0 | 157 (131.7-196.3)  −4.4 | 163 (16.4)  −2.9 | 161 (131.7-196.3)  −4.2 |
| **Post dose 1.5 h**  **Change from predose** | 159 (17.7)  −7.2 | 158 (130.0-194.3)  −7.1 | 160 (16.1)  −5.9 | 160 (129.0-191.3)  −5.6 |
| **Post dose 2 h**  **Change from predose** | 159 (17.0)  −7.3 | 158 (127.3-192.3)  −6.8 | 160 (16.4)  −6.0 | 160 (128.7-199.3)  −4.3 |
| **Post dose 3 h**  **Change from predose** | 160 (16.6)  −6.4 | 158 (128.3-191.7)  −5.1 | 158 (15.5)  −8.0 | 159 (123.3-186.3)  −7.2 |
| **Post dose 4 h**  **Change from predose** | 162 (18.3) ^a^  −5.2 | 158 (126.3-211.3)^a^  −4.8 | 159 (15.1)  −6.5 | 161 (126.7-186.7)  −7.8 |
| **Post dose 6 h**  **Change from predose** | 157 (17.8)  −9.9 | 155 (126.7-196.7)  −8.9 | 156 (16.9)  −9.5 | 157 (122.0-194.3)  −9.5 |
| **Post dose 8 h**  **Change from predose** | 158 (18.2)  −8.1 | 158 (123.7-201.0)  −6.3 | 156 (15.0)  −9.5 | 156 (120.0-194.7)  −8.6 |
| **Post dose 12 h**  **Change from predose** | 159 (18.0)  −7.5 | 157 (121.3-195.7)  −6.7 | 159 (17.2)  −7.1 | 157 (124.7-191.0)  −6.9 |
| **Post dose 24 h**  **Change from predose** | 164 (19.3)  −2.4 | 163 (128.7-205.0)  −1.9 | 163 (18.2)  −3.3 | 162 (130.3-203.3)  −2.4 |

| **QRS (msec)** | **Momelotinib 200 mg**  **(N=48)** | | **Momelotinib 800 mg**  **(N=48)** | |
| --- | --- | --- | --- | --- |
|  | **Mean (SD)** | **Median (range)** | **Mean (SD)** | **Median (range)** |
| **Predose** | 94.7 (8.4) | 93.6 (79.0-126.3) | 94.8 (7.7) | 95.0 (81.0-116.1) |
| **Post dose 0.5 h**  **Change from predose** | 95.1 (8.6) ^a^  0.5 | 92.7 (82.7-123.0) ^a^  0.2 | 96.3 (8.3) ^a^  1.3 | 96.3 (82.7-116.0) ^a^  0.9 |
| **Post dose 1 h**  **Change from predose** | 95.4 (8.7) ^a^  0.8 | 95.0 (82.0-117.7) ^a^  0.7 | 94.2 (8.8)  −0.5 | 93.0 (76.7-116.7)  −0.2 |
| **Post dose 1.5 h**  **Change from predose** | 93.6 (9.2)  −1.1 | 92.2 (70.3-122.7)  −0.8 | 94.4 (7.9)  −0.3 | 94.3 (82.0-116.7)  0.2 |
| **Post dose 2 h**  **Change from predose** | 94.2 (8.3)  −0.5 | 93.5 (77.0-119.0)  −1.0 | 93.2 (8.5)  −1.6 | 91.8 (80.3-118.3)  −1.3 |
| **Post dose 3 h**  **Change from predose** | 93.4 (8.2) ^a^  −1.3 | 92.0 (76.0-119.7) ^a^  −1.6 | 93.4 (8.4)  −1.4 | 92.2 (78.7-117.0)  −1.2 |
| **Post dose 4 h**  **Change from predose** | 91.7 (8.7)  −3.0 | 89.3 (73.0-120.3)  −2.3 | 93.5 (9.5)  −1.3 | 92.5 (75.7-117.3)  −1.9 |
| **Post dose 6 h**  **Change from predose** | 93.2 (8.4)  −1.5 | 91.2 (81.0-117.0)  −1.5 | 93.5 (8.7)  −1.3 | 92.0 (78.7-119.3)  −1.1 |
| **Post dose 8 h**  **Change from predose** | 92.6 (8.5)  −2.1 | 91.0 (76.7-117.0)  −2.1 | 93.0 (9.4)  −1.8 | 90.8 (78.3-117.0)  −1.6 |
| **Post dose 12 h**  **Change from predose** | 93.8 (8.5)  −0.9 | 93.3 (77.0-118.7)  −0.6 | 94.2 (9.2)  −0.6 | 93.8 (79.7-119.3)  −0.2 |
| **Post dose 24 h**  **Change from predose** | 92.9 (9.3)  −1.8 | 92.2 (69.3-123.7)  −1.6 | 94.2 (8.4)  −0.6 | 93.0 (79.0-118.7)  −0.4 |

| **QRS (msec)** | **Placebo**  **(N=48)** | | **Moxifloxacin 400 mg**  **(N=48)** | |
| --- | --- | --- | --- | --- |
|  | **Mean (SD)** | **Median (range)** | **Mean (SD)** | **Median (range)** |
| **Predose** | 94.9 (8.3) | 93.9 (78.2-124.3) | 94.1 (8.6) | 92.9 (76.9-117.3) |
| **Post dose 0.5 h**  **Change from predose** | 95.4 (8.1)  0.5 | 94.8 (79.0-118.3)  0.7 | 95.1 (9.4)  1.0 | 93.5 (77.3-119.7)  1.6 |
| **Post dose 1 h**  **Change from predose** | 95.1 (9.4)  0.2 | 94.8 (77.7-121.7)  −0.1 | 93.6 (8.4)  −0.5 | 93.3 (79.0-116.0)  −0.4 |
| **Post dose 1.5 h**  **Change from predose** | 94.2 (8.5)  −0.7 | 92.3 (80.3-119.3)  −0.2 | 93.6 (9.6)  −0.5 | 93.0 (74.7-120.7)  −0.3 |
| **Post dose 2 h**  **Change from predose** | 93.2 (8.8)  −1.7 | 92.3 (77.7-116.3)  −1.0 | 92.7 (9.3)  −1.4 | 90.7 (73.7-118.3)  −0.9 |
| **Post dose 3 h**  **Change from predose** | 92.6 (8.4)  −2.3 | 91.5 (77.0-117.3)  −2.2 | 92.5 (8.6)  −1.6 | 91.8 (74.7-117.7)  −1.2 |
| **Post dose 4 h**  **Change from predose** | 93.3 (7.9) ^a^  −1.5 | 92.0 (76.0-116.7) ^a^  −0.8 | 91.9 (9.3)  −2.2 | 88.2 (78.0-118.0)  −0.9 |
| **Post dose 6 h**  **Change from predose** | 92.6 (8.2)  −2.3 | 91.7 (74.7-117.7)  −2.4 | 92.4 (9.0)  −1.7 | 90.2 (76.7-115.3)  −1.6 |
| **Post dose 8 h**  **Change from predose** | 93.7 (8.8)  −1.2 | 92.7 (79.0-115.7)  −0.9 | 92.6 (8.8)  −1.5 | 92.2 (75.0-119.7)  −1.1 |
| **Post dose 12 h**  **Change from predose** | 93.7 (8.4)  −1.2 | 93.7 (77.3-113.3)  −0.2 | 93.3 (8.8)  −0.8 | 92.8 (79.0-118.0)  −0.4 |
| **Post dose 24 h**  **Change from predose** | 93.7 (8.4)  −1.2 | 92.8 (80.0-117.0)  −0.6 | 92.5 (8.9)  −1.6 | 92.5 (75.0-119.0)  −0.7 |

| **RR (msec)** | **Momelotinib 200 mg**  **(N=48)** | | **Momelotinib 800 mg**  **(N=48)** | |
| --- | --- | --- | --- | --- |
|  | **Mean (SD)** | **Median (range)** | **Mean (SD)** | **Median (range)** |
| **Predose** | 962 (132.9) | 949 (714.1-1440.7) | 967 (131.2) | 956 (756.1-1443.7) |
| **Post dose 0.5 h**  **Change from predose** | 835 (128.2) ^a^  −126.6 | 812 (586.3-1262.7) ^a^  −111.1 | 826 (134.6) ^a^  −140.4 | 785 (667.0-1318.3) ^a^  −125.3 |
| **Post dose 1 h**  **Change from predose** | 813 (129.9) ^a^  −148.6 | 782 (591.0-1264.7) ^a^  −152.1 | 785 (119.0)  −181.7 | 770 (602.0-1143.3)  −165.1 |
| **Post dose 1.5 h**  **Change from predose** | 803 (113.43)  −159.0 | 795 (529.3-1110.0)  −161.0 | 771 (126.2)  −195.8 | 756 (486.0-1029.0)  −164.4 |
| **Post dose 2 h**  **Change from predose** | 801 (113.1)  −161.0 | 765 (593.7-1084.3)  −159.7 | 771 (115.6)  −196.1 | 761 (538.0-1043.3)  −165.2 |
| **Post dose 3 h**  **Change from predose** | 826 (127.4)  −136.8 | 785 (606.0-1119.0)  −124.4 | 781 (105.3)  −185.6 | 784 (608.3-1022.3)  −173.3 |
| **Post dose 4 h**  **Change from predose** | 837 (129.6)  −125.1 | 810 (542.0-1122.3)  −109.0 | 789 (130.2)  −178.2 | 768 (546.7-1083.0)  −172.1 |
| **Post dose 6 h**  **Change from predose** | 808 (119.4)  −153.5 | 815 (625.7-1178.3)  −138.9 | 770 (124.0)  −196.9 | 752 (572.7-1104.7)  −176.3 |
| **Post dose 8 h**  **Change from predose** | 836 (139.9)  −126.0 | 831 (603.0-1289.3)  −108.6 | 788 (115.9)  −179.1 | 767 (546.3-1055.3)  −175.9 |
| **Post dose 12 h**  **Change from predose** | 780 (118.5)  −181.7 | 745 (618.3-1140.7)  −174.8 | 745 (97.5)  −221.6 | 735 (603.0-1020.0)  −231.3 |
| **Post dose 24 h**  **Change from predose** | 864 (128.4)  −98.0 | 841 (651.7-1141.3)  −91.6 | 828 (114.6)  −138.5 | 800 (568.3-1187.7)  −125.1 |

| **RR (msec)** | **Placebo**  **(N=48)** | | **Moxifloxacin 400 mg (N=48)** | |
| --- | --- | --- | --- | --- |
|  | **Mean (SD)** | **Median (range)** | **Mean (SD)** | **Median (range)** |
| **Predose** | 946 (133.5) | 919 (727.6-1342.8) | 956 (130.1) | 959 (740.9-1476.9) |
| **Post dose 0.5 h**  **Change from predose** | 826 (113)  −120.2 | 813 (598.7-1182.3)  −113.8 | 846 (121.2)  −112.4 | 830 (630.7-1247.0)  −105.8 |
| **Post dose 1 h**  **Change from predose** | 827 (120.7)  −119.5 | 807 (645.0-1227.3)  −126.6 | 826 (140.5)  −132.2 | 808 (587.3-1309.7)  −132.7 |
| **Post dose 1.5 h**  **Change from predose** | 830 (127.9)  −116.0 | 820 (586.7-1230.7)  −101.9 | 836 (141.7)  −122.1 | 813 (610.0-1387.0)  −112.9 |
| **Post dose 2 h**  **Change from predose** | 847 (129.8)  −98.9 | 831 (626.0-1336.0)  −98.7 | 851 (133.2)  −107.7 | 818 (565.0-1323.3)  −107.8 |
| **Post dose 3 h**  **Change from predose** | 904 (151.1)  −42.4 | 879 (642.3-1394.0)  −31.6 | 889 (112.6)  −69.9 | 864 (611.0-1201.7)  −49.3 |
| **Post dose 4 h**  **Change from predose** | 915 (149.1) ^a^  −29.6 | 890 (661.7-1358.0) ^a^  −24.9 | 910 (121.6)  −48.2 | 881 (708.3-1237.7)  −30.2 |
| **Post dose 6 h**  **Change from predose** | 843 (127.9)  −103.7 | 829 (639.7-1356.7)  −119.2 | 837 (109.1)  −121.9 | 819 (606.3-1189.7)  −91.5 |
| **Post dose 8 h**  **Change from predose** | 872 (130)  −74.3 | 875 (681.3 1369.0)  −60.2 | 859 (130.2)  −99.6 | 839 (645.0-1404.7)  −81.2 |
| **Post dose 12 h**  **Change from predose** | 841 (139.9)  −105.2 | 837 (629.7-1321.3)  −98.7 | 826 (105.7)  −132.4 | 802 (635.3-1219.7)  −136.1 |
| **Post dose 24 h**  **Change from predose** | 894 (140.8)  −52.0 | 867 (669.3-1337.0)  −42.3 | 886 (110.4)  −72.7 | 871 (712.7-1162.3)  −66.7 |

| **HR (msec)** | **Momelotinib 200 mg**  **(N=48)** | | **Momelotinib 800 mg**  **(N=48)** | |
| --- | --- | --- | --- | --- |
|  | **Mean (SD)** | **Median (range)** | **Mean (SD)** | **Median (range)** |
| **Predose** | 63.2 (8.2) | 62.9 (41.4-83.8) | 62.8 (8.0) | 62.6 (41.1-78.9) |
| **Post dose 0.5 h**  **Change from predose** | 73.0 (10.8) ^a^  9.8 | 73.3 (47.0-102.3)  7.9 | 73.9 (10.5) ^a^  11.0 | 76.0 (45.0-90.0)  10.2 |
| **Post dose 1 h**  **Change from predose** | 75.1 (10.4) ^a^  11.8 | 76.7 (47.0-101.3)  12.9 | 77.7 (11.3)  14.9 | 77.5 (52.0-99.3)  12.7 |
| **Post dose 1.5 h**  **Change from predose** | 75.8 (10.7)  12.6 | 75.0 (53.7-113.0)  12.3 | 79.9 (14.8)  17.1 | 78.8 (58.0-123.3)  12.8 |
| **Post dose 2 h**  **Change from predose** | 76.0 (10.4)  12.7 | 78.2 (55.0-100.7)  12.4 | 79.2 (12.2)  16.4 | 78.5 (57.0-111.3)  15.5 |
| **Post dose 3 h**  **Change from predose** | 73.9 (11.1) ^a^  10.7 | 76.0 (53.3-98.7)  10.6 | 77.9 (10.6)  15.1 | 76.2 (58.3-101.0)  14.0 |
| **Post dose 4 h**  **Change from predose** | 73.1 (11.8)  9.9 | 74.0 (53.3-110.3)  8.6 | 77.8 (12.9)  15.0 | 78.0 (55.3-109.3)  14.8 |
| **Post dose 6 h**  **Change from predose** | 75.3 (10.5)  12.1 | 73.5 (50.3-95.3)  11.5 | 79.5 (12.2)  16.6 | 79.7 (54.0-104.3)  13.8 |
| **Post dose 8 h**  **Change from predose** | 73.3 (11.6)  10.1 | 71.8 (46.0-99.3)  8.9 | 77.4 (11.3)  14.6 | 78.0 (56.7-109.3)  13.7 |
| **Post dose 12 h**  **Change from predose** | 78.1 (10.7)  14.8 | 80.3 (52.3-96.3)  15.1 | 81.4 (10.2)  18.6 | 81.2 (58.3-99.3)  19.6 |
| **Post dose 24 h**  **Change from predose** | 70.7 (10.2)  7.4 | 71.3 (52.7-92.0)  6.7 | 73.5 (9.9)  10.7 | 74.7 (50.0-105.0)  10.0 |

| **HR (msec)** | **Placebo**  **(N=48)** | | **Moxifloxacin 400 mg (N=48)** | |
| --- | --- | --- | --- | --- |
|  | **Mean (SD)** | **Median (range)** | **Mean (SD)** | **Median (range)** |
| **Predose** | 64.4 (8.4) | 65.1 (44.3-82.7) | 63.5 (8.0) | 62.3 (40.8-80.8) |
| **Post dose 0.5 h**  **Change from predose** | 73.5 (9.6)  9.1 | 73.5 (50.3-99.7)  9.2 | 71.8 (9.5)  8.3 | 71.8 (47.3-95.0)  7.7 |
| **Post dose 1 h**  **Change from predose** | 73.6 (10.1)  9.2 | 73.8 (48.3-92.7)  8.8 | 74.2 (11.6)  10.7 | 73.8 (45.3-107.0)  10.1 |
| **Post dose 1.5 h**  **Change from predose** | 73.5 (10.7)  9.1 | 72.7 (48.3-102.3)  8.9 | 73.2 (11.0)  9.7 | 73.3 (42.7-97.7)  8.9 |
| **Post dose 2 h**  **Change from predose** | 71.8 (9.8)  7.5 | 71.7 (44.3-95.7)  7.4 | 71.7 (10.3)  8.2 | 73.0 (45.0-105.7)  8.5 |
| **Post dose 3 h**  **Change from predose** | 67.7 (10.8)  3.3 | 67.8 (42.5-95.0)  2.1 | 68.3 (9.08.95)  4.8 | 69.2 (49.7-97.7)  3.8 |
| **Post dose 4 h**  **Change from predose** | 66.8 (10.3) ^a^  2.3 | 67.7 (43.7-91.7) ^a^  1.7 | 66.6 (8.7)  3.2 | 67.8 (48.3-85.3)  1.9 |
| **Post dose 6 h**  **Change from predose** | 72.3 (9.9)  7.9 | 72.2 (44.3-93.0)  7.6 | 72.5 (9.2)  9.0 | 72.7 (50.0-98.3)  6.3 |
| **Post dose 8 h**  **Change from predose** | 69.7 (9.4)  5.4 | 68.5 (43.3-87.7)  3.4 | 70.9 (9.7)  7.4 | 72.0 (42.0-92.3)  6.8 |
| **Post dose 12 h**  **Change from predose** | 72.6 (10.9)  8.2 | 71.3 (45.3-95.0)  7.1 | 73.3 (8.5)  9.8 | 74.3 (49.0-94.0)  9.7 |
| **Post dose 24 h**  **Change from predose** | 68.2 (9.5)  3.9 | 68.8 (44.5-89.3)  3.2 | 68.5 (8.1)  5.0 | 68.8 (51.0-85.3)  5.1 |

^a^ N=47.

**Table S6. Momelotinib statistical analysis of dose proportionality**

| PK parameter | GLSM | | GLSM ratio | 90% CI |
| --- | --- | --- | --- | --- |
|  | Momelotinib 200 mg (N=48) | Momelotinib 800 mg (N=48) |  |  |
| Momelotinib | | | | |
| AUC_inf_ (h• ng/mL) | 3690 | 8812 | 239 | 221-258 |
| AUC**_last_** (h• ng/mL) | 3426 | 7904 | 231 | 214-249 |
| C_max_ (ng/mL) | 465 | 839 | 180 | 167-194 |
| M21 | | | | |
| AUC_inf_ (h• ng/mL) | 3985 | 8964 | 225 | 210-241 |
| AUC**_last_** (h• ng/mL) | 3443 | 7477 | 217 | 203-232 |
| C_max_ (ng/mL) | 389 | 685 | 176 | 163-190 |

Statistical analysis for dose proportionality was based on the mixed-effect model, including sequence, period, and treatment as fixed effects, and participants within sequence as a random effect.

AUC_inf_, area under the curve from zero to infinity; AUC_last_, area under the curve from zero to the last measurable concentration; CI, confidence interval; C_max_, maximum plasma concentration; GLSM, geometric least squares mean; PK, pharmacokinetic.

**Table S7. Moxifloxacin single-dose pharmacokinetic parameters^a^**

| PK parameter | Moxifloxacin 400 mg  (N=48) |
| --- | --- |
| AUC_inf_ (h• ng/mL), mean (%CV) | 40937 (20.0) |
| AUC**_last_** (h• ng/mL), mean (%CV) | 31587 (20.7) |
| C_max_ (ng/mL), mean (%CV) | 2602 (24.7) |
| t_1/2_ (h), mean (SD) | 10.8 (1.8) |
| T_max_ (h), median (Q1,Q3) | 3.0 (2.0, 4.0) |

AUC_inf_, area under the curve from zero to infinity; AUC_last_, area under the curve from zero to the last measurable concentration; C_max_, maximum plasma concentration; PK, pharmacokinetic; t_1/2_, terminal elimination half-life; T_max_, time to achieve maximum plasma concentration.

^a^ From the momelotinib pharmacokinetic analysis set.

**Table S8. Statistical analysis of the relationship between momelotinib plasma concentration and time-matched, baseline-adjusted, and placebo-controlled QTcF**

|  | | | 95% CI | |  |
| --- | --- | --- | --- | --- | --- |
|  | Estimate | Standard error | Lower | Upper | *P* value |
| Overall regression^a^ |  |  |  |  |  |
| Intercept (a) | −0.544 | 0.883 | −2.32 | 1.23 | .541 |
| Concentration (b) | −0.00207 | 0.000993 | −0.00402 | −0.000119 | .038 |
| Regression with sex as fixed effect^b^ |  |  |  |  |  |
| Intercept (a) | −1.09 | 1.32 | −3.74 | 1.56 | .413 |
| Concentration (b) | −0.00208 | 0.000994 | −0.00403 | −0.000127 | .037 |
| Sex (c): 0 = male; 1 = female | 0.939 | 1.67 | −2.42 | 4.30 | .577 |

CCHG_QTcF, time-matched, baseline-adjusted, and placebo-corrected QTcF; CI, confidence interval; PK, pharmacokinetic; QTcF, corrected QT interval using the Fridericia formula.
^a^ Overall regression equation was CCHG_QTcF = a + (b × concentration). Overall PK/pharmacodynamic regression included concentration as a continuous covariate and participant within sequence as a random effect. ^b^ Regression equation with sex as a fixed effect was CCHG_QTcF = a + (b × concentration) + (c × sex). PK/pharmacodynamic regression with sex as a fixed effect included sex as a fixed effect, concentration as a continuous covariate, and participant as a random effect.

**Table S9. Summary of AEs**

| n (%) | Momelotinib 200 mg  (N=48) | Momelotinib  800 mg  (N=48) | Placebo  (N=48) | Moxifloxacin  400 mg  (N=48) |
| --- | --- | --- | --- | --- |
| TEAE | 12 (25) | 18 (37.5) | 6 (12.5) | 6 (12.5) |
| Treatment-related AE | 10 (20.8) | 12 (25.0) | 1 (2.1) | 2 (4.2) |
| TEAE by SOC and preferred terms | | | | |
| Nervous system disorders | 7 (14.6) | 12 (25.0) | 2 (4.2) | 1 (2.1) |
| Headache | 5 (10.4) | 10 (20.8) | 1 (2.1) | 1 (2.1) |
| Dizziness | 2 (4.2) | 6 (12.5) | 1 (2.1) | 0 |
| Facial nerve disorder | 0 | 1 (2.1) | 0 | 0 |
| Paresthesia | 0 | 1 (2.1) | 0 | 0 |
| Gastrointestinal disorders | 3 (6.3) | 10 (20.8) | 1 (2.1) | 2 (4.2) |
| Nausea | 1 (2.1) | 8 (16.7) | 0 | 0 |
| Vomiting | 1 (2.1) | 4 (8.3) | 0 | 0 |
| Chapped lips | 0 | 2 (4.2) | 0 | 1 (2.1) |
| Aphthous stomatitis | 0 | 0 | 0 | 1 (2.1) |
| Constipation | 0 | 1 (2.1) | 0 | 0 |
| Diarrhea | 0 | 0 | 1 (2.1) | 0 |
| Soft feces | 0 | 1 (2.1) | 0 | 0 |
| Gastritis | 1 (2.1) | 0 | 0 | 0 |
| Gastroesophageal reflux disease | 1 (2.1) | 0 | 0 | 0 |
| Skin and subcutaneous tissue disorders | 1 (2.1) | 3 (6.3) | 1 (2.1) | 3 (6.3) |
| Rash pruritic | 1 (2.1) | 1 (2.1) | 0 | 1 (2.1) |
| Contact dermatitis | 0 | 1 (2.1) | 0 | 1 (2.1) |
| Acneiform dermatitis | 0 | 1 (2.1) | 0 | 0 |
| Macular rash | 0 | 0 | 0 | 1 (2.1) |
| Xeroderma | 0 | 0 | 1 (2.1) | 0 |
| Vascular disorders | 1 (2.1) | 7 (14.6) | 0 | 0 |
| Flushing | 1 (2.1) | 6 (12.5) | 0 | 0 |
| Hypotension | 0 | 1 (2.1) | 0 | 0 |
| Reproductive system and breast disorders | 2 (4.2, 10.0^a^) | 1 (2.1, 5.0^a^) | 1 (2.1, 5.0^a^) | 2 (4.2, 10.0^a^) |
| Metrorrhagia | 2 (4.2, 10.0^a^) | 1 (2.1, 5.0^a^) | 0 | 2 (4.2, 10.0^a^) |
| Breast tenderness | 1 (2.1) | 0 | 0 | 0 |
| Dysmenorrhea | 1 (2.1, 5.0^a^) | 0 | 0 | 0 |
| Menorrhagia | 0 | 0 | 1 (2.1, 5.0^a^) | 0 |
| Cardiac disorders | 0 | 3 (6.3) | 0 | 0 |
| Palpitations | 0 | 2 (4.2) | 0 | 0 |
| Sinus tachycardia | 0 | 1 (2.1) | 0 | 0 |
| General disorders and administration site conditions | 1 (2.1) | 3 (6.3) | 0 | 0 |
| Asthenia | 1 (2.1) | 2 (4.2) | 0 | 0 |
| Chills | 0 | 1 (2.1) | 0 | 0 |
| Infections and infestations | 1 (2.1) | 1 (2.1) | 1 (2.1) | 0 |
| Body tinea | 1 (2.1) | 0 | 0 | 0 |
| Otitis externa | 0 | 1 (2.1) | 0 | 0 |
| Urinary tract infection | 0 | 0 | 1 (2.1) | 0 |
| Musculoskeletal and connective tissue disorders | 1 (2.1) | 1 (2.1) | 1 (2.1) | 0 |
| Back pain | 0 | 1 (2.1) | 0 | 0 |
| Muscle spasm | 1 (2.1) | 0 | 0 | 0 |
| Pain in extremity | 0 | 0 | 1 (2.1) | 0 |
| Respiratory, thoracic, and mediastinal disorders | 0 | 1 (2.1) | 0 | 0 |
| Hyperventilation | 0 | 1 (2.1) | 0 | 0 |
| Eye disorders | 1 (2.1) | 0 | 0 | 0 |
| Vision blurred | 1 (2.1) | 0 | 0 | 0 |

AE, adverse event; SOC, system organ class; TEAE, treatment-emergent adverse event.

^a^ Percentage based on female participants (n=20).

References

1. Nakagawa S, Johnson PCD, Schielzeth H. The coefficient of determination R(2) and intra-class correlation coefficient from generalized linear mixed-effects models revisited and expanded. *J R Soc Interface.* 2017;14(134).
